# Evaluation of GAN-Based Model for Adversarial Training

**DOI:** 10.3390/s23052697

**Published:** 2023-03-01

**Authors:** Weimin Zhao, Qusay H. Mahmoud, Sanaa Alwidian

**Affiliations:** Department of Electrical, Computer and Software Engineering, Ontario Tech University, Oshawa, ON L1G 0C5, Canada

**Keywords:** neural network, deep learning, adversarial samples, adversarial training, image classification

## Abstract

Deep learning has been successfully utilized in many applications, but it is vulnerable to adversarial samples. To address this vulnerability, a generative adversarial network (GAN) has been used to train a robust classifier. This paper presents a novel GAN model and its implementation to defend against L_∞_ and L_2_ constraint gradient-based adversarial attacks. The proposed model is inspired by some of the related work, but it includes multiple new designs such as a dual generator architecture, four new generator input formulations, and two unique implementations with L_∞_ and L_2_ norm constraint vector outputs. The new formulations and parameter settings of GAN are proposed and evaluated to address the limitations of adversarial training and defensive GAN training strategies, such as gradient masking and training complexity. Furthermore, the training epoch parameter has been evaluated to determine its effect on the overall training results. The experimental results indicate that the optimal formulation of GAN adversarial training must utilize more gradient information from the target classifier. The results also demonstrate that GANs can overcome gradient masking and produce effective perturbation to augment the data. The model can defend PGD L_2_ 128/255 norm perturbation with over 60% accuracy and PGD L_∞_ 8/255 norm perturbation with around 45% accuracy. The results have also revealed that robustness can be transferred between the constraints of the proposed model. In addition, a robustness–accuracy tradeoff was discovered, along with overfitting and the generalization capabilities of the generator and classifier. These limitations and ideas for future work will be discussed.

## 1. Introduction

Deep learning models have exhibited their extensive capabilities in multiple domains of application. However, generalization is one of the challenges related to the current deep learning models. More specifically, our modern deep learning model failed to generalize on some manually crafted data samples. These data samples are generally referred to as adversarial samples. Normally, adversarial samples can be obtained by adding small, computed perturbations to the existing data sample. From a human perspective, these small vectors are meaningless and insignificant; however, they can cause our deep learning models to behave in an unexpected manner. The existence of the adversarial samples means that the current deep learning model cannot generalize consistently across all the data distributions; this is related to the security of the deep learning models, especially regarding some safety-critical systems.

The algorithm used to generate adversarial samples is called adversarial attacks. There are currently multiple methods to construct the attack algorithms (e.g., gradient methods, constraint methods, or metaheuristic methods) [1,2,3,4,5]. The general goal of the adversarial attack algorithm is to optimize a data sample toward a direction that maximizes the loss value of the model. Some more well-known algorithms are the gradient-based attack algorithms that exploit the gradient of the loss of the models and the backpropagation process to generate the adversarial samples [1,2]. This paper focuses on proposing a solution to improve the robustness of the convolution classifier against these gradient-based attack algorithms.

Most attack algorithms use distance metrics to measure the adversarial sample’s differences from the original test sample. Commonly used metrics for image classification adversarial samples include L_0_, L_2_, and L_∞_ metrics, in which attack algorithms typically have a constraint term in the optimization formulation to limit the maximum difference of the generated adversarial samples. The L_0_ metric measures the number of value differences by comparing the adversarial sample to the original sample, and L_∞_ measures the maximum value changes across all the sample values, whereas the L_2_ metric measures the Euclidean distance of the adversarial distortion. In gradient-based attacks, L_2_ and L_∞_ metrics are commonly used [1,2]; however, the L_0_ metric is widely used in other types of attack algorithms [4]. This paper addresses the problems relevant to gradient-based attacks using the L_2_ and L_∞_ distance metrics.

Adversarial training is a state-of-the-art defense method against adversarial attacks [6,7]. This method works by adding adversarial samples to the mix with the original training data samples during the training to improve the model’s robustness against adversarial samples. In addition to adversarial training, a Generative Adversarial Network (GAN) is an alternative training strategy that can provide similar robustness. The current state-of-the-art GAN defensive frameworks use two major methodologies to mitigate adversarial perturbation. One general methodology involves constructing a generative model to transfer the distribution of the data that maps the adversarial sample to another data distribution to mitigate the effect of adversarial perturbation [8,9,10,11,12,13,14,15,16]. However, we are more interested in using GAN for direct adversarial sample generation and data augmentation, as it forms a similar formulation to adversarial training. This type of GAN is easier to implement and train in terms of overall complexity compared to the transformation models. Previous work [17,18,19] utilized GAN to augment the training samples to address the adversarial sample problem. In this paper we address the following challenges induced by gradient-based attacks in adversarial training and GAN adversarial augmentation frameworks:

- Conventional adversarial training may require an iterative gradient-based attack algorithm to augment the training data and to guarantee the model’s robustness. This process heavily increases the training complexity. Using GAN rather than attack algorithms could reduce the training complexity, since it generally requires less backpropagation times. While previous works have implemented GAN for adversarial training, this paper proposes new extensions to reduce the training complexity.
- The gradient masking problem for gradient-based attack algorithms under the L_2_ setting renders attack algorithms less effective and reduces the conventional adversarial training performance to train a robust model. This norm constraint is challenging to implement in a GAN-based training framework and is not achievable by using the activation function.
- Previous GAN models have not utilized different possible formulations of GAN. The new formulations introduced in this paper provide more insights into building an optimal GAN for adversarial training purposes.

In this paper, we introduce a GAN-based model to augment the training sample under both L_∞_ and L_2_ constraints, with improvements to L_∞_ constraint augmentation. Furthermore, we included the L_2_ constraint, since this constraint is less frequently considered by the augmentation-style GAN model in adversarial machine learning, and it is necessary to address this gap. We address the problems related to these gradient-based attacks within the adversarial training process by utilizing generative adversarial networks (GANs) to generate adversarial samples and to augment the training data. It is also interesting to generate adversarial samples using the GAN method, and we believe it could enable a greater understanding of the adversarial samples. To this end, the contributions of this paper are:

- A GAN framework to reduce the training complexity of conventional adversarial training.
- A GAN-based model architecture to improve deep learning convolution classifiers’ adversarial robustness against L_∞_ and L_2_ constrained gradient-based attacks. The proposed GAN’s generators can also learn to produce effective L_∞_ and L_2_ adversarial samples against the co-trained classifier model, and it is less affected by the gradient masking problem compared to other gradient-based attacks in the L_2_ setting.
- Evaluation of different formulations and hyperparameters of the proposed GAN-based model for data augmentation and adversarial robustness improvement.

The novelty of the proposed GAN model includes a dual generator design, the implementation of multiple generator input vectors, and the implementation to provide augmentation on both L_∞_ and L_2_ constraint. In addition, the limitations of the proposed GAN-based training are identified and discussed, including the accuracy tradeoffs, overfitting, and the limited generalization capabilities. This paper extends preliminary research results presented in [5].

The rest of the paper is organized as follows. Section 2 discusses the related work regarding gradient-based attacks and constraints and introduces adversarial training and the defensive methods related to GANs. Section 3 introduces the methodologies and formulations of our framework. Section 4 presents our experimental setups, evaluations, and discussions. Finally, Section 5 includes the conclusion and discussion of future works.

## 2. Related Works

This section introduces the general concept of gradient-based adversarial attacks. It also discusses the related works regarding adversarial training and defensive GAN models under adversarial machine learning settings.

### 2.1. Gradient Attacks

The gradient-based attacks algorithm has evolved in different variations [7]. However, this section only discusses the most important ones and the algorithm used for our evaluation. We believe that this is sufficient to understand the works in this paper.

Goodfellow et al. [1] found that some small vectors could easily alter the output results of the deep learning model when the vectors are added to corresponding input samples. This phenomenon persists even when the vector is multiplied by a small scalar value. This type of vector was obtained by using a one-step backpropagation gradient descent on a selected deep learning model, and it represented the loss function’s gradient of the deep learning model within the input space. Subsequently, this method has been referred to as the Fast Gradient Sign Method (FGSM) with the following formulation:

(1) X’=X+Ɛ·sign(∇x L(x, y))

where *X* is the input sample, *X’* is the adversarial sample, ∇*x L*(*x*, *y*) represents the loss gradient regarding the input data *x*, and *Ɛ* is the constraint norm size of the distortion. Furthermore, Goodfellow et al.’s [1] research revealed that the inclusion of a sign operator can improve the effectiveness of the algorithm in attacking the model.

Madry et al. [2] found that computing only one loss gradient was insufficient to find a worst-case adversarial sample that maximizes the loss of the model. The Projected Gradient Descent (PGD) is an improved method built upon FGSM to enable iterative computation over the loss gradient of the model. The PGD formula could be written as follows:

(2) $$X_{t+1}=\prod_{x+s}\left(X’+\alpha \;sign\left(\nabla x\;L\left(x,\;y\right)\right)\right)$$

where we compute the next step of the adversarial sample *X_t_*_+1_ based on the previous step sample *X’*. Each step uses a similar operation relative to FGSM to obtain the gradient information ∇*x L*(*x*, *y*) of the current sample. The overall perturbation vector is also constrained by a defined norm size, and the out-of-bound vector is projected back into the feasible constraint.

**Figure 1:**
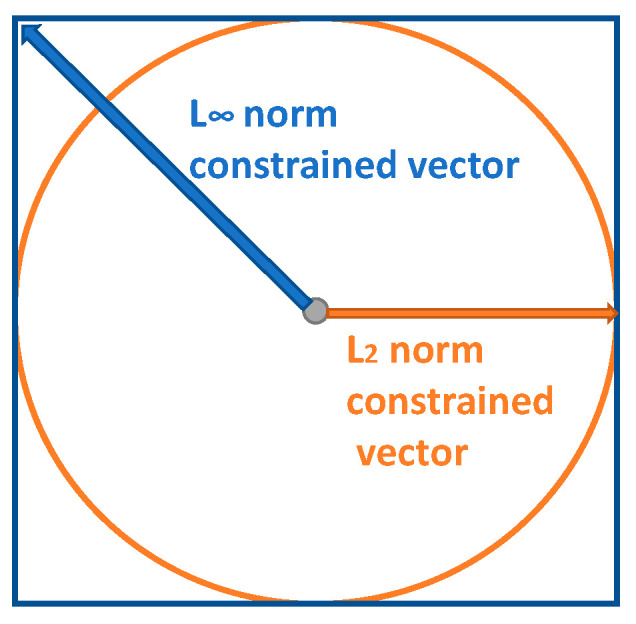
L_2_ norm and L_∞_ norm comparison.

Furthermore, the attack algorithm can be implemented under different constraints [2,7]. The L_∞_ constraint is widely used for attacking image datasets that constrain the maximum perturbed pixel value across all the pixels in the target image. The L_2_ constraint limits the maximum Euclidean distance of the adversarial sample to the original sample. The L_0_ constraint limits the maximum number of pixels that could be perturbed by the algorithm; however, it is less utilized in the gradient-based attacks. The different constraint algorithms provide different adversarial sample solutions to attack the deep learning model. Figure 1 illustrates the L_2_ and L_∞_ norm vectors in a 2D space. The center point indicates a data sample point with (*x*, *y*) value. The arrows are the L_2_ and L_∞_-constrained vectors with the same norm value but with different constraints. The points located on the orange circle have the same L_2_ distance to the center point, and the points on the blue square have the same L_∞_ distance to the center point. An L_∞_-constrained vector could be larger in value if it is measured with an L_2_ metric, resulting a different sample distribution when different constraint vectors are added to an identical data sample. Researchers have typically considered the L_∞_ constraint with gradient-based attacks since it is more effective in attacking deep learning models and keeps the perturbation relatively invisible. Furthermore, the L_2_ constraint gradient attacks have a problem with gradient masking [2]; therefore, it is less effective in uncovering hidden adversarial samples.

### 2.2. Adversarial Training

Goodfellow et al. [1] introduced a method called adversarial training to augment the training data using an attack algorithm to improve the model’s robustness against adversarial perturbation. The optimization formulation is written as follows:

(3) $$\min\sum \max L\left(f\left(x_{i}+\delta \right),\;y_{i}\right)$$

where the framework includes the adversarial sample *x_i_ + δ*, which maximizes the loss of the model *f* regarding the data label *y_i_*. The outer optimization minimizes the maximum loss to make the model *f* generalize on the adversarial sample. The vector *δ* is the adversarial perturbation vector. Madry et al. [2] claimed that this is an effective method for training a robust model. The state-of-the-art adversarial training uses iterative-based attacks, such as the PGD algorithm, to generate adversarial samples for augmentation purposes during the training process. Madry et al. [2] suggested that this is necessary to train a robust model against a strong attack, such as the PGD attack. In general, the final robustness of the model after adversarial training depends upon whether the attack used for data augmentation is effective and sufficient to find the worst-case adversarial samples. Therefore, multi-iteration attacks are recommended for adversarial training. However, these attack algorithms also introduce additional iterations of back-propagation during the training process and increase the training complexity.

### 2.3. GAN Models

The state-of-the-art generative models have achieved exceptional performance in terms of image generation [20], style transformation [21], and other applications. In adversarial machine learning communities, generative models have also been proposed to defend against adversarial attacks. All GAN models include some form of min-max optimization, which can be summarized as follows:

(4) $$\min\sum \max L\left(f\left(G)\right),\;y_{i}\right)$$

where classifier *f* minimizes the loss on generator *G*. Generator *G* maximizes the loss of *f*. Overall, any GAN includes the component networks of the generator and discriminator to complete the formulation. We propose that the basic min-max formulation of GAN (4) is similar to the conventional adversarial training (3); however, GAN may involve more complex learning objectives.

Some recent research [8,9,10,11,12,13,14,15,16] has utilized the GAN model as a cleanse transformation model to map the adversarial distribution to the normal data distribution. These models usually utilize an auto-encode-decoder structure network to realize the data transformation. Various studies [8,9,10] have implemented a generator to train against adversarial samples from attack algorithms to realize the cleansing function. The generator minimizes the classifier’s loss by updating its own parameters to provide a valid denoise function. Laykaviriyakul et al. [11] also implemented a bidirectional GAN that learns to transfer the sample between the adversarial and normal distribution. The other models [12,13,14,15,16] utilize an unsupervised learning strategy for sample reconstruction. The generator of these GANs is responsible for reconstructing any input sample from a latent vector to realize the defense function. The reconstruction process can mitigate the adversarial noise of adversarial samples. Another implementation of GAN involves using them as an alternative data augmentation method to replace or enhance the attack algorithm during the adversarial training process [17,18,19]. Liu et al. [17] proposed a GanDef framework that treats the classifier as a latent vector generator. The latent vector generated from the classifier is discriminated by the discriminator network to output a Boolean vector, which indicates whether the latent vector is related to an adversarial sample. The classifier can train against this discriminator to reduce the discriminative loss when it encounters an adversarial sample input. Another GAN framework [18] utilized the formulation to control a noise injection module for robust optimization. The generator in this framework reconstructs the sample from the classifier’s latent outputs, and the discriminator detects and limits the distribution of the reconstructed sample to prevent over-perturbation. Wang et al. [19] used GAN to facilitate a more direct approach. The generator in this GAN framework is implemented as the threat model that learns to maximize the classifier’s loss. The classifier then trains against the adversarial samples from the generator to provide generalization on general adversarial samples. This approach of GAN involves a more simplified structure and replaces the multi-iteration attack algorithm from conventional adversarial training with a generator model. Between the related strategies, conventional adversarial training is the standard baseline method that can provide reliable robustness improvement to a deep learning classifier model. However, it requires extensive training, complexity, and additional knowledge of attacks for successful implementation. The GAN cleanse model can realize convenient attachment to any classifier model. However, it generally requires additional model deployments and cannot provide robustness to original classifiers. Some of them also suffer from more intensive training complexity. The performances of these models are also related to the detailed implementation of the GAN parameters and architectures. The GAN augmentation can be used to enhance the quality of adversarial training or to provide a simplified augmentation architecture that reduces the training complexity or implementation requirements of the adversarial training. However, it shares a limitation of other GAN models, namely that the performance is related to the GAN formulation and parameter settings. The description and limitations are summarized in Table 1.

This work evaluates GAN’s ability to facilitate adversarial data augmentation, similar to Wang et al.’s paper [19]. The reason we selected this GAN augmentation strategy is that it involves a simple architecture that can be easily modified and extended to provide adversarial robustness to different types of classifier models in the future. We aimed to capitalize on the benefits of the formulation and propose our modified model with multiple sub-versions to enhance and evaluate robustness. The proposed model involves a dual generator design to reduce potential overfitting and provide more regularization toward the generalization. The subversions include four proposals of generator input vector formulations and two implementations under L_∞_ and L_2_ constraints. In the next section, four different formulations of generators are discussed to explore the superior method to construct an effective GAN model for adversarial robustness. These different formulations are first evaluated in the L_∞_ norm constraint since it is a well-studied constraint. Furthermore, more research is discussed that evaluates other hyperparameters under the L_∞_ norm. The optimal formulation and hyperparameter setting are further tested with the L_2_ constraint. We believe that L_2_ norm perturbation is a more challenging formulation for GAN, since there is no restriction on each dimension value of the input. The normal method of L_∞_ constraints, such as clipping and using the “tanh” soft-clip function, does not work in this case. Hence, it necessitates another means to constrain the generator model’s output to fit the L_2_ constraint. Aside from these generation issues, we also considered the benefits of GAN to remove the multi-iteration attack algorithm in adversarial training to reduce the training complexity. Additionally, we found that our GAN network can be highly effective in finding the adversarial perturbation with our constraint settings, which mitigate the gradient masking issue. However, it is worthwhile noting that our GAN cannot avoid the common limitations of adversarial training and the conventional GAN, such as limitations related to generalization, accuracies tradeoffs, and overfitting problems. These limitations are discussed in Section 4.

## 3. Methodology

This section introduces our proposed solution. The subsections delineate the overall architecture, components used for evaluation, training procedures, and loss functions. The overall architecture subsection describes our proposed GAN formulation and the dual generator architecture. The components of the GAN include the input of the generator, different constraints of generator implementation, and a brief introduction to the classifier architecture. They are described in corresponding subsections. The training procedure covers the training methodology of the GAN model and provides information about the generator and the classifier parameters’ update process. This subsection also applies a training complexity analysis to compare the proposed GAN training and conventional adversarial training. During the implementation of the GAN, the execution time of the training method is affected by multiple factors, such as model sizes, the execution of other background programs, and training environments. Hence, rather than providing an exact execution time, we provide this complexity analysis to demonstrate when our solution is more advantageous relative to conventional adversarial training. Finally, the general descriptions of the loss functions are provided in the last subsection.

### 3.1. Overall Architecture

**Figure 2:**
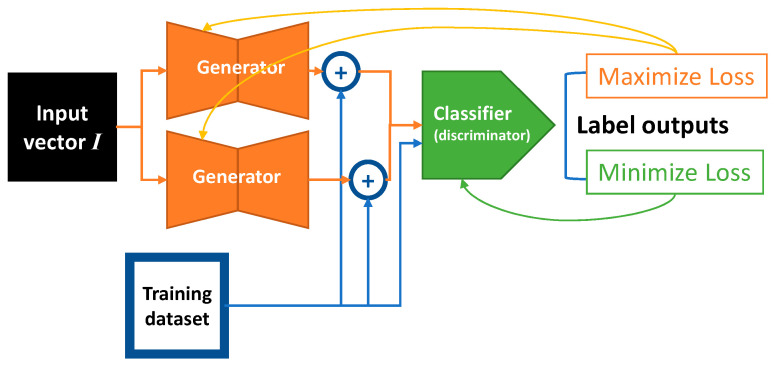
The architecture of our proposed GAN.

The overall proposed architecture is consistent with a traditional GAN model; however, we implemented the generator to find the perturbation vector that can maximize the loss of our discriminator. In this case, our discriminator is the classifier we wish to train to perform accurate and robust classification on our test dataset. In the subsequent section, we will refer to this discriminator as the classifier. The formulation of our proposed GAN optimization is the following:

(5) $$min_{D}max_{G}\sum L\;\left(D\left(x_{i}\right),\;y_{i}\right)+\sum L\left(D\left(x_{i}+\varepsilon G\left(I\right)\right),\;y_{i}\right)$$

where *x_i_* is the training sample and *y_i_* is the corresponding label. The formulation represents an optimization goal that generator *G* provides the perturbation *ƐG*(*I*) added to the original sample *x_i_*, which maximizes the loss of classifier *D*. The value *Ɛ* is a scalar to constrain the maximum norm of perturbation. There are L_∞_ and L_2_ norm constraint measurements to be considered in this paper. Hence, we implemented two methods for generator *G* to output the perturbation vector. The scalar value *Ɛ* is used in both L_∞_ and L_2_ constraints with different implementations, which is discussed in the following subsections. Classifier *D* minimizes the loss with the sample perturbed by the generator and the original sample *x_i_*. *I* is an input vector of generator *G*. To improve the stability of the model, we implemented a dual generator architecture, which means that there are two generators, *G*, that simultaneously produce adversarial perturbation. The goal of the dual generator architecture is to provide more regularization toward our proposed GAN. This architecture is suggested by Im et al. [22], as it is beneficial to include more generator discriminator pairs to produce more diverse results. The high-level architecture is illustrated in Figure 2. The perturbation vectors generated from both generators are added with training samples to produce adversarial samples used for training the classifier model. The yellow arrows within Figure 2 indicate the backpropagation of the gradient to maximize the classifier loss. The green arrow indicates the backpropagation of the gradient to minimize the classifier loss. 

One of the objectives of this paper is to evaluate the different formulations and parameters of our proposed GAN to provide information regarding the possible direction of improvement. Hence, there are four major paper sections of evaluation to assess generator input formulations—GAN, training epochs, L_2_ robustness, and the transferability of constraint robustness. To help understand these evaluation processes, we will discuss several important components and their implementation in specific subsections. The important components include the input vector *I*, the generator *G*, and the classifier *D* from the GAN formulation (5), as well as the training procedure and the loss function. 

### 3.2. Generator Input Formulation

We proposed four different implementations of *I* to formulate four generators for evaluation. These four implementations of *I* will be compared to conclude which one is a more viable choice to produce a better training result. The following are the different formulations regarding the input vector *I*:

(6) $$I_{1}=x$$

(7) $$I_{2}=Concatenate\left(x,z\right)$$

(8) $$I_{3}=sign\left(\nabla L\left(x\right)\right)$$

(9) $$I_{4}=Concatenate\left(x,\;sign\left(\nabla L\left(x\right)\right)\right)$$

The descriptions of these input vectors are follows:

- (6): The input vector is the original data sample.
- (7): The input vector is the combination of the original data sample and a normally distributed latent noise vector.
- (8): The input vector is the loss gradient direction (signed gradient) of the target classifier regarding the input sample *x* and current parameters. We will refer to (8) as *sign*(∇) in later sections.
- (9): The input vector is the combination of the original data sample and the loss gradient direction. We will refer to (9) as *Concatenate*(*x, sign*(∇)) in later sections.

The signed gradient vector is computed by using the FGSM attack algorithm to perform one-step backpropagation. This process requires one additional backpropagation and does not significantly impact the training complexity. These four generator input formulations are evaluated in L_∞_ constraint settings since this constraint is well-studied. The optimal formulation is selected for further testing.

### 3.3. Generator Implementation for L_∞_ Constraint Perturbation

The generator *G* within our proposed GAN architecture is responsible for generating a constraint adversarial vector. The output of the generator can be written as follows:

(10) O=ε G(I)

In the L_∞_ constraint, the scalar *Ɛ* limits the value range of the output vector *O*. In this case, the “tanh” soft-clip function can limit every output value within the range of [−1,1] with a properly defined gradient. Hence, the “tanh” function is implemented in this constraint generator. If we expand the formulation of the L_∞_ constraint generator, it can be written as follows:

(11) $$O=\varepsilon \;G\left(I\right)=\varepsilon \;tanh\left(f_{\theta }\left(I\right)\right)$$

where *O* is the output perturbation vector and *Ɛ* is the scalar value. *f_θ_* is the inner generator neural network model, and *I* is one of the selected input vectors. In this case, the output of “tanh” is limited to the [−1,1] range, and the scalar *Ɛ* can be set as any real number to adjust the upper and lower bound of the values.

### 3.4. Generator Implementation for L_2_ Constraint Perturbation

**Figure 3:**
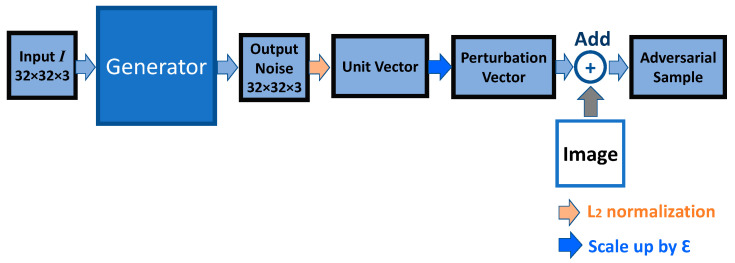
The overview of the L_2_ generator process.

In the L_2_ constraint setting, we applied another approach to limit the maximum length of the output vector. We applied L_2_ normalization, scale-up, and an addition to the generated output vector to construct the final adversarial sample for data augmentation. Figure 3 illustrates the high-level process of the generator.

**Figure 4:**
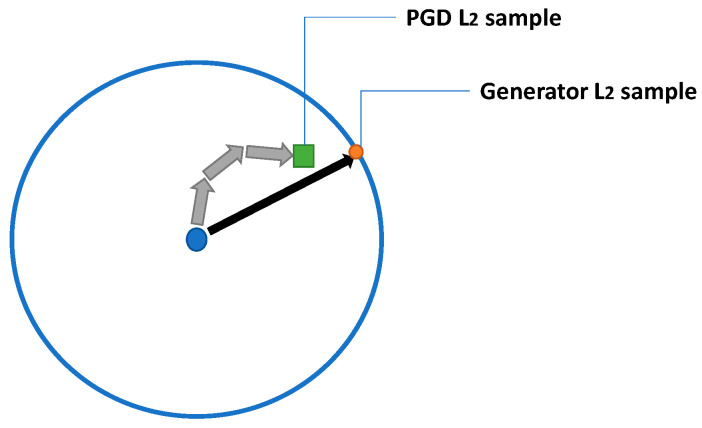
The difference between our generator’s sample and the PGD sample. The blue circle represents the L_2_ ball around the sample.

In this case, we implemented L_2_ normalization to normalize every output vector into a unit vector and used a scalar *Ɛ* to scale up the perturbation vector. The expansion of the formulation can be written as follows:

(12) $$O=\varepsilon \;G\left(I\right)=\varepsilon \;{\frac{f_{\theta }\left(I\right)}{\left|\left|f_{\theta }\left(I\right)\right|\right|}}_{2}$$

where we first compute a unit vector of the generator’s neural network output *f_θ_* and scale the unit vector’s length with the scaler *Ɛ*. In this case, the neural network model *f_θ_* is only responsible for finding the direction of the perturbation, and this unit vector is forced to have a length consistent with the L_2_ constraint value *Ɛ*. The additional benefit is that it reduces the difficulty of determining the exact perturbation for the generator, as the generator is not required to produce exact accurate pixel values. We believe that by using this method, the generator can escape the masked gradient, since the output norm length is always maximized. However, the limitation is that our generator invariably produces a vector that has a length of maximum constraint value. This means that it could only find the adversarial solutions located on the L_2_ ball around the target sample. The difference between our generator and PGD is illustrated in Figure 4. It illustrates that PGD can find the solutions inside the L_2_ ball, and our generator cannot find these solutions.

### 3.5. Classifier Architecture

The classifier we implemented is a CNN model with a similar structure to the VGG model [23]. The model has an input of the image dimension and an output of the label dimension. We reduced the size of the standard VGG to fit the scope of the project. The detailed parameters are discussed in the experiment section, along with our dataset.

### 3.6. Training Procedure

**Figure 5:**
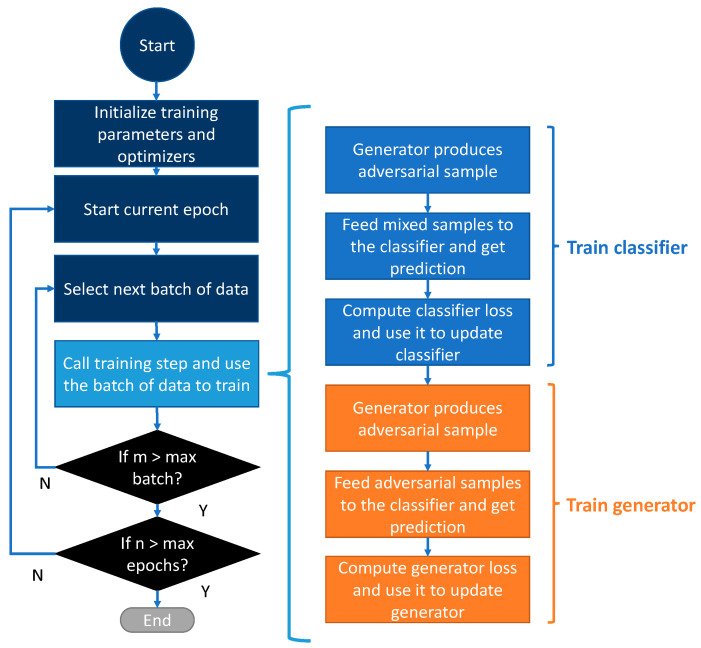
Training procedure.

This section presents the general training procedure of our proposed GAN. The training process of the GAN includes two loop functions. The outer loop function is responsible for fetching the batch of the training samples and controlling the maximum training epochs. When all the training samples are fetched, the outer loop functions enter the subsequent epochs. The inner loop function uses the fetched batch of data to perform optimization and to update the model parameters. Each complete update is referred to as one training step. Within one training step, the classifier and generator parameters are updated in a sequence. In the first part of the training step, the generator will first produce the adversarial samples for training, and the adversarial samples will be mixed with clean samples and fed into the classifier to calculate the classification loss. The parameters of the classifier are then updated based on this loss. In the second part, the generator reproduces the adversarial samples based on the updated classifier. The new adversarial samples are then fed into the classifier to calculate the new loss values. This new loss is used to update the generator parameters. The training procedure is illustrated in Figure 5.

In addition, we have performed a training complexity analysis. The training complexity of the proposed method is O (n × m × k) in big-O notation, where n is the total epoch value, m is the total training step within one epoch, and k represents the backpropagation requirements within one training step. The k value of our purposed GAN is the summarized value of the adversarial sample generation time (if required) and the total network parameter update time within one training step. When the input formulation (8) or (9) is used, one backpropagation is required for the FGSM algorithm to generate input gradients. Hence, the worst-case k value is 5, which includes two FGSM calculations and three parameter updates within one training step. The overall training complexity is O (5 × n × m). Assuming a conventional adversarial training process, the same big-O evaluation is applied with O (n × m × k). The k value is the summarized value of backpropagation time, which includes the model parameter update time and the gradient descent time used for the attack algorithm within one training step. The attack algorithm is used to produce *δ* in formula (3). To provide valid augmentation, the gradient descent time for attacks is normally large. The big-O of adversarial training is larger than the proposed model when k is greater than 4, such that O ((4 + 1) × n × m) = O (5 × n × m).

### 3.7. Loss Functions

The loss function of the classifier is the categorical cross-entropy loss. With the generator, we aim to maximize the cross-entropy loss; hence, we multiply the cross-entropy loss with a value of −1 and apply it to the generator’s loss function. Furthermore, the optimizer and regularization methods are discussed in the experiment section, along with the other experimental settings.

## 4. Experiments and Results

The dataset used for this experiment is the Canadian Institute For Advanced Research (CIFAR) 10 dataset, with 32 × 32 × 3 as image dimensions and 10 categories. The dataset has been provided by the TensorFlow library. We used the default 50,000 training samples and 10,000 testing samples for training and evaluation, correspondingly. Since the classification task of the CIFAR 10 dataset is multiclass classification, the accuracy metric is used for reflecting the overall performance of the classification and the adversarial robustness. The accuracy metric utilizes the following calculation formula:

(13) $$\mathrm{Accuracy}=\frac{\mathrm{TP}+\mathrm{TN}}{\mathrm{TP}+\mathrm{FP}+\mathrm{TN}+\mathrm{FN}}$$

where the TP, TN, FP, and FN represent true positive, true negative, false positive, and false negative. The accuracy metric is represented as the overall percentage of true classification results over the total samples.

**Figure 6:**
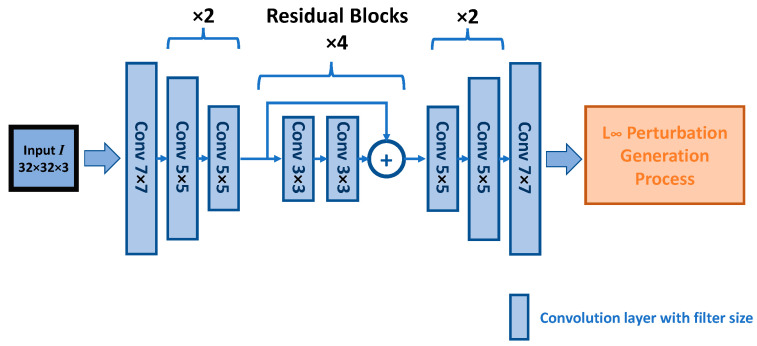
L_∞_ constraint generator architecture.

**Figure 7:**
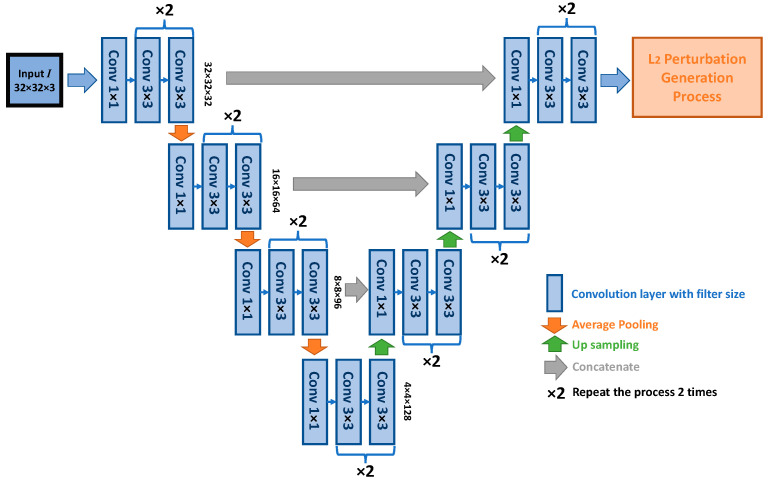
L_2_ constraint generator architecture.

We have two sets of model structures for the two different constraint types. The overall structures and functionalities are similar. Under both constraints, the generator inputs a 32 × 32-dimensional vector (the channel depends on the selected input formulation) and outputs a 32 × 32 × 3-dimensional vector. The structure implementation of the L_∞_ constraint generator is a cycle GAN [21] style generator with a residual network backbone, and the structure implementation of the L_2_ constraint generator is a u-net architecture. Generally, these two types of generator architectures can yield similar results. However, during our extensive experiment, we selected each architecture with slightly better performance for each constraint. Their parameter settings and the diagrams are presented in Table 2, Table 3 and Table 4, Figure 6 and Figure 7, including the classifier parameters. We reported the detailed parameters, including the output dimension and the filter settings from each layer of the model. The filter settings include three sets of values for each layer. These values represent the kernel sizes, filter numbers, and stride sizes. In Table 2 and Table 3, the stride size is omitted, since we did not use convolution as the down and up sample method; hence, the stride size is always one by one for these models. Some layers’ parameters may ultimately include an “× value”, meaning that we repeated the layer a certain number of times in sequence. In addition, we also applied different “width” values to the L infinity constraint generator. The width value is a multiplier that scales up the filter numbers for some models. The details of the width are discussed in the subsequent sections. Aside from the generator differences, the norm sizes established for each constraint setting depend upon the training performance. Generally, the L_∞_ constraint adversarial sample is more challenging for a classifier to generalize on; thus, the distortion norm sizes we used under this constraint are lower in value compared to the L_2_ norm constraint. The details are discussed in subsequent sections, with corresponding results.

In the classifier model, we applied the dropout regularization layer for each layer. The first two convolution layers’ dropout value was set to 0.3, and the rest of the layers’ dropout was 0.4. Additional data augmentation was used before the images were fed into the training process, including “random switches” with a range of [0, 0.1] and “random flipped”.

The Adam optimizer was used for training our proposed GAN model. The learning rate for the classifier is 0.0001, with 0.5 beta_1_ and 0.9 beta_2_. The learning rate for the generator is 0.0002, with 0.5 beta_1_ and 0.9 beta_2_. We observed that the lower learning rate of the classifier could improve the stability of the proposed GAN and that the generator can produce more consistent adversarial perturbation solutions.

An additional benchmark model was built to compare the performances. The model architecture is consistent with the classifier with the same parameter settings. The benchmark model has been introduced as the baseline model in our experiments.

The attack algorithms included in the evaluation are FGSM, PGD, and Carlini and Wagner L_2_ (CW-L_2_) attacks. The PGD has been used to evaluate adversarial robustness in both constraints; FGSM provides a comparison in contrast to PGD in L_∞_ constraint experiments; and the CW-L_2_ attack is another adversarial attack that uses the constraint optimization method. It is used to provide additional evaluation for L_2_ robustness. The maximum iteration parameters of PGD attacks were set to 100 and 1000, depending on the different experiments. Additional information is discussed in later sections since we used different settings for evaluation.

In subsections, the different formulations of generators are experimented with and evaluated with L_∞_ constraint. The optimal formulation of the generator is used for further experiments to evaluate the impact of the training epoch and to test its performance with L_2_ constraint settings. We conducted our experiment in this order, since the L_∞_ adversarial noise is more commonly used with the gradient-based attack algorithms and is usually less affected by the gradient masking problem [2]. Hence, they are more concerned and effective in fooling the classifier compared to their L_2_ counterparts. A more systematic evaluation of the L_∞_ constraint setting is preferred.

In each experiment, we have adhered to the following process for evaluation. First, we implemented different GAN frameworks with different settings according to the evaluation content of the experiment. These settings include generator input, generator constraint, generator width, generator scalar value, generator architecture, and training epochs. The implemented GANs will be trained using the proposed training procedure. Subsequently, the trained classifiers will be evaluated by corresponding attack algorithms in different experiments. The attack algorithm will be determined with specific parameter settings, including attack type, constraint, norm sizes, and iteration numbers. Finally, we have recorded the accuracy of each classifier regarding the attack algorithms’ settings and reported the result with plots and tables. The details of these GAN and attack settings are presented in each subsection for each experiment. 

### 4.1. L_∞_ Generator Formulations

**Figure 8:**
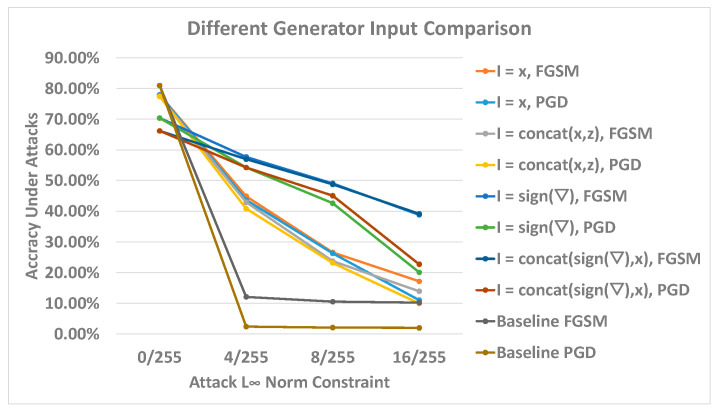
Generators’ formulations comparison. The *x*-axis represents the adversarial perturbation norm sizes, and the *y*-axis illustrates the model’s accuracies under different norm sizes.

This section evaluates the proposed methods to train robust classifiers against the L_∞_ constraint gradient attacks. The first step of our evaluation involved verifying the different formulations of input proposed in Section 3. We trained four different classifiers with the four formulations with 100 epochs. The width of the generator was set to a value of “1”, and the scalar value was set to 16/255 to constrain the generator L_∞_ norm output vector. The evaluation uses FGSM and PGD attacks to generate adversarial samples based on the test data set. The L_∞_ norms include 4/255, 8/255, and 16/255 to test the classifier robustness under different adversarial perturbation strengths. The results are plotted in Figure 8.

The results plot indicates a clear pattern within the accuracy trends. We identified roughly three groups of behavior regarding the model’s accuracy and robustness. The baseline model has an accuracy above 80%; however, the robust accuracies under all attacks decline to around 10% for FGSM and 0% for PGD. The models trained with the input of *x* and *concatenate*(*x*,*z*), which do not include any gradient information, have slightly less clean accuracies. The robust accuracies start from above 40% at 4/255 attack strength and decay to between 10% to 20% at 16/255 attack strength for all attack settings. For these models, the FGSM accuracies are slightly higher than the PGD accuracies. However, they do not have substantial differences, indicating that the models gain similar adversarial robustness against both FGSM and PGD. The gradient landscape of these models became simpler so that the one-step gradient descent could find a similar adversarial sample to the adversarial sample found by 100-step gradient descent. However, the shape of the landscape does not have a definitive relationship with the robustness; hence, the robust accuracy of these models is low. The third group of the models includes all of the classifiers trained with the generator with its gradient direction (input formulation of *sign*(∇) and *concatenate*(*x*,*sign*(∇))). These models have significantly less clean accuracies but have more robustness improvement across all attack settings compared to other groups. Regarding the robust accuracies, this group of classifiers has a larger deviation of values when comparing the FGSM and PGD accuracies. The largest accuracy value difference is at 16/255 norm size; specifically, the FGSM accuracies remain around 40%, and the PGD accuracies decline to just above 20%. However, all of the FGSM and PGD accuracies remain above other groups. Additionally, within this group, the *sign*(∇) model classifier has slightly less robust accuracies compared to the *concatenate*(*x*,*sign*(∇)) model; however, the *concatenate*(*x*,*sign*(∇)) model has a larger clean accuracy downgrade. Overall, they exhibit no significant advantage between the two formulations.

The experimental results conclusively indicate that our proposed GAN architecture can improve adversarial robustness. The plot pattern demonstrates that the robustness improvement is related to the generator input formulation and the generator’s capability to estimate the adversarial samples. The GAN involving the generator with the classifier’s one-step gradient information performs better in overall settings and can be considered a more optimal setup. We suggest that accessing the current gradient direction can help the generator discover worst-case adversarial perturbation similar to what PGD can do; thus, the classifier that is adversarially trained with this generator can generalize better to the adversarial sample. However, the limitation persists that the highest robust accuracy under the 16/255 PGD attack is only around 20%, which is not sufficient to be an accurate classification result. The clean and robust accuracy trade-off is also presented in the result, and it is difficult to improve robustness without a significant clean accuracy drop. Furthermore, all GAN frameworks we trained have a significant overfitting effect regarding the classifier. The reported clean data testing accuracies achieve accuracy levels between 65% and 80%, but the clean training accuracies can reach over 90%. In contrast, the baseline classifier has both training and testing accuracies above 81% with clean data. We tested most of the regularization techniques, including L_2_ regularization or Adam with weight decay (AdamW). However, the results did not improve. More catastrophic problems arose when the weight decay value was above 0.0001. We speculate that the generated adversarial sample has a shifted distribution so that the classifier cannot generalize on both original and shifted data.

### 4.2. L_∞_ Training Epochs

**Figure 9:**
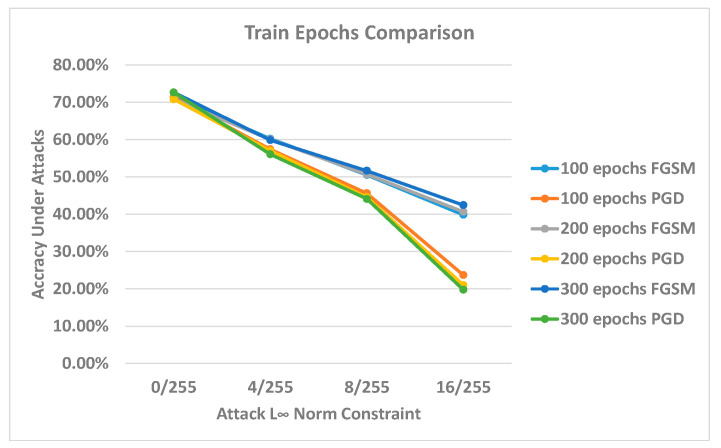
Training epoch comparison. The *x*-axis represents the adversarial perturbation norm sizes, and the *y*-axis indicates the models’ accuracies under different norm sizes.

This section represents the classifiers’ accuracy relationship to the number of training epochs. According to the last section, we selected the optimal performance formulations regarding the clean and robust accuracy for this experiment. The selected formulation is the *sign*(∇) model. Furthermore, we increased the width of the generator to “2” to increase its capability. We found that this width value can increase the performance of the result by a small margin. The PGD 100 iteration attack has been used for this experiment. The results of the experiment are illustrated in Figure 9.

We started with the previous training epochs first, with 100 training epochs. We treated this as a standard training epoch value. Compared to the standard 100 epochs, the results for 200 and 300 epochs are similar. However, with more attack strength, the accuracy difference between the different training epoch models increases. The 16/255 results exhibit the most significant accuracy difference. The FGSM accuracy slightly increases with increased training epochs, indicating that more training steps can improve the model’s robustness against this single-step gradient attack. However, the PGD accuracy begins to decrease with more epochs. We suggest that this phenomenon constitutes a sign of overfitting regarding the generator. With more training epochs, the generator overfits into the one-step gradient direction and fails to discover a new and more effective adversarial direction. The problem is commonly discovered in adversarial machine learning models related to the gradient saturation effect that can penalize the learning result [24]. One other possible speculation is that the adversarial training between the generator and the classifier becomes excessively dynamic, as the classifier pursues the generator to fit on the current generated data distribution and deviates from the previously fit distribution; however, the generator can invariably find a new adversarial direction. As a result, there is always an adversarial direction for the classifier, and the equilibrium point is difficult to reach. 

### 4.3. L_∞_ PGD 1000

The PGD 100 iterations are applied for the previous section for the purpose of rapid evaluation. However, to improve the creditability, we have included PGD 1000 iterations to validate the results. We used the same model from the last section and applied PGD 1000 to evaluate whether the accuracy deviates from the previous results. Table 5 presents the comparison.

We did not observe a significant deviation of accuracies when comparing the two different iteration settings. The increasing iteration of attacks exerted a low impact on model performance with all norm sizes. We proposed that the PGD 100 attack is sufficient for our models to confirm the previous accuracy performances.

### 4.4. L_2_ Robustness

**Figure 10:**
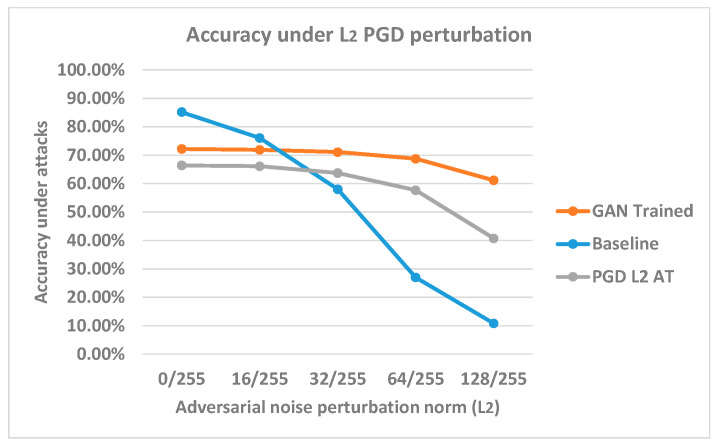
Accuracy comparison under L_2_ PGD attack. The *x*-axis represents the adversarial perturbation norm sizes, and the *y*-axis indicates the model’s accuracies under different norm sizes.

This section evaluates the model robustness under L_2_ perturbations. The scalar value used for the generator is 64/255 with the L_2_ setting, and the classifier is trained with 100 epochs with our proposed GAN framework. The *sign*(∇) is used for generator input formulation. In this experiment, the L_2_ norm size is set as 0/255, 16/255, 32/255, 64/255, and 128/255 for the PGD attack to evaluate the model performance with the test dataset. In L_2_ settings, we considered including larger norm values to evaluate the robustness because the L_2_ constraint adversarial sample was not effective for small norm sizes with gradient-based attacks in our experiments, and the gradient attack may suffer from the gradient masking problem. The increasing norm size can be helpful to leverage the problem. For the same reason, for the L_2_ constraint setting, we applied PGD 1000 for all evaluations and removed the FGSM attack. The benchmark model was used for comparison. Additionally, we trained another model with an identical structure to the benchmark model using conventional adversarial training. We have included this extra model for L_2_ constraint experiments, since the gradient masking [2] problem is known for L_2_ gradient attacks, and we have used this model to test and compare the performance of regular L_2_ gradient attack adversarial training and our GAN training method with L_2_ constraint. In this adversarial training process, PGD L_2_ was used with a 64/255 constraint value to augment the training data. The results are plotted in Figure 10. The adversarially trained model is named after PGD L_2_ AT in the plot.

For the benchmark model with no defenses, the clean data accuracy is around 85%, and the accuracy drops to around 10% with 128/255 norm size. In our GAN-trained classifier model, we found that the clean accuracy is lower than the benchmark model, at around 70% accuracy. The accuracy becomes closer for the two models under the 16/255 L_2_ perturbation size. When a larger perturbation is added, the GAN-trained model remains relatively consistent in terms of accuracy compared to the baseline and begins to decline slightly more when the distortion is larger than 64/255. This result suggests that our GAN-trained model significantly improves the robustness against L_2_ PGD attacks; however, a robustness–clean accuracy trade-off exists. This type of trade-off was also described in previous research [25]. The model adversarially trained with PGD L_2_ attack also retains a higher accuracy under all perturbation norm sizes; however, the performance is worse than our GAN-trained model. We conclude that the proposed GAN training can be more effective than PGD adversarial training under the L_2_ constraint setting.

In a further experiment, we applied our trained generator to generate adversarial samples against our GAN-trained classifier using the test dataset. We found that the performance of the generator was superior in finding the adversarial perturbation with the L_2_ constraint compared to PGD L_2_ attacks. The comparison results are presented in Table 6. However, we have also implemented an attack algorithm that optimizes box-constraint and CW-L_2_ attack, and the results indicate that the model is not robust under this attack method. This result reveals that more adversarial samples exist close to the test data samples in the Euclidean distance measurement. The robustness evaluated by PGD L_2_ may not reflect the true L_2_ robustness of the classifier because of the gradient masking, which was discussed in previous research [2]. Despite this result, our generator’s attacking performance is close to the CW-L_2_ attack on generating L_2_ constraint adversarial samples. Hence, we believe that GAN is an effective tool for generating adversarial perturbation compared to normal L_2_ gradient-based methods and that it is less affected by the gradient masking effect. The reason that the adversarial sample still exists for our current GAN-trained classifier most likely relates to our classifier’s capability to generalize on the samples rather than the generator’s precision.

### 4.5. L_∞_ PGD Robustness on L_2_-Trained Model

**Figure 11:**
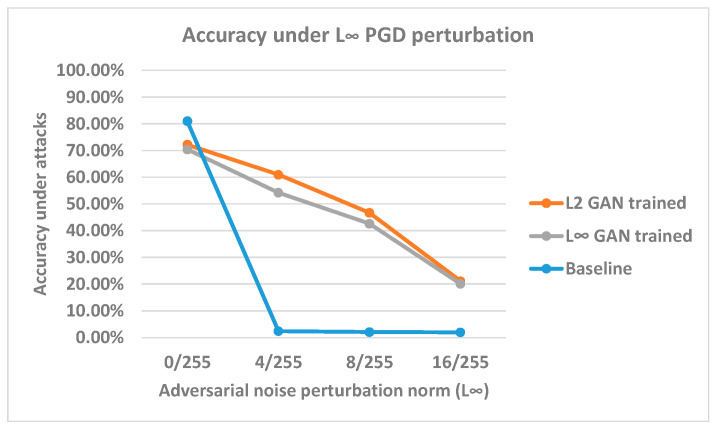
Accuracy under PGD L_∞_ attack. The *x*-axis represents the adversarial perturbation norm sizes, and the *y*-axis illustrates the model’s accuracies under different norm sizes.

In addition, we have also run PGD in the L_∞_ setting to test how our GAN-trained model augmented with L_2_ perturbation could perform. A model trained under one norm constraint typically cannot guarantee robustness against the adversarial sample in another constraint. This experiment tests whether our L_2_ GAN-trained classifier can also be robust against an L_∞_ constraint PGD attack. We used 0/255, 4/255, 8/255, and 16/255 as the norm sizes, as these are normally used in the L_∞_ norm constraint. The results are plotted in Figure 11.

Under the L_∞_ norm constraint PGD attack, the performance of our model is significantly inferior compared to the PGD L_2_ accuracies. The accuracy declines to 20% with a 16/255 L_∞_ norm size. The PGD L_∞_ perturbation is less affected by the gradient masking issue and more effective in attacking our model. Hence, an inferior performance is expected. Nonetheless, the model still performs better than the benchmark model. This result indicates that training against L_2_ perturbation still enhances robustness against L_∞_ adversarial samples. Furthermore, the robustness of the L_2_ trained model and the L_∞_ trained model do not differ substantially from each other, which indicates that our L_2_ generator can still provide valid augmentation to L_∞_ robustness. However, we note that we used a substantially larger scalar size in the L_2_ setting (64/255) compared to the L_∞_ setting (16/255) to achieve similar effects.

### 4.6. Visualization

This section provides a visualization of the generated adversarial noises and the pre-SoftMax output of the model in the discovered adversarial direction. We have compared the adversarial distortion noise generated from different sources (FGSM, PGD, CW-L_2_, and our generator) to describe different features and to compare their effects on the output of the model. 

**Figure 12:**
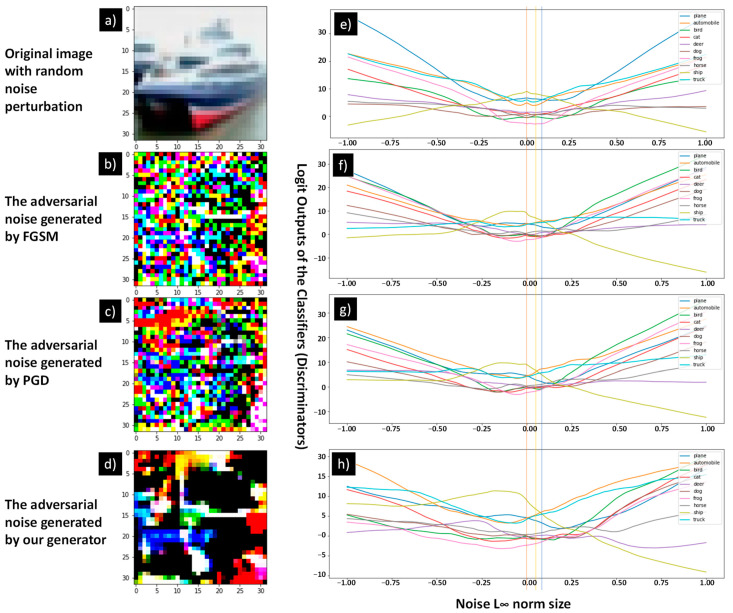
L_∞_ adversarial noise visualization with the model’s logit outputs. The left-hand side presents the figures of the original image (**a**), adversarial noise from the FGSM attack (**b**), adversarial noise from the PGD attack (**c**), and adversarial noise from our generator (**d**). The right-hand side contains the pre-SoftMax output values trending when the original image is perturbated by a random noise (**e**), the FGSM adversarial noise (**f**), the PGD adversarial noise (**g**), and the generator adversarial noise (**h**). The labels of the right-hand side plots are shared with *x* and *y* axes.

The first group of comparison is the model from the L_∞_ setting experiments. The model we have opted to visualize is the GAN, with *sign*(∇) as the input formulation and “2” as the width parameter. We have also selected the one trained with 300 epochs to determine the maximum effect of our GAN training. The FGSM and PGD adversarial distortion noises have also been generated based on this model. Figure 12 presents the visualization.

Image (a) is our selected example image with the correct label of “ship”. Image (b), (c) and (d) represent the generated adversarial perturbation noise from the algorithms FGSM, PGD, and our generator, respectively. All the noises are renormalized to have their values between [−1,1] to standardize the L_∞_ norm size. The plots (e), (f), (g), and (f) correspond to the pre-SoftMax logit outputs of the classifier when the image (a) is perturbed by different norm sizes of the following:

1. Zero mean gaussian noise with a standard deviation of one,
2. FGSM perturbation (b),
3. PGD perturbation (c),
4. and generator perturbation (d).

The *x*-axis of the plots indicates the amount of norm size by which we modified the noise and added onto the image (a), and the *y*-axis represents the output value of the classifier. The relationship can be described as follows:

(14) O=f(x+ϵ δ)

where *x* is the original image vector, *ϵ* is the norm multiplier represented in the *x*-axis, and *δ* is the noise vector. *f* is the selected classifier, and *O* is the output of *f*, which is the *y*-axis value. The detail of the gaussian noise is not displayed, since there is no significant information within the gaussian noise, and it represents the model’s normal behavior within a noisy environment.

The plot indicates that under a regular gaussian noise, the output of the model regarding the norm size produces a symmetrical shape, which means that in normal circumstances, the model is consistent with *ϵ* increasing or decreasing in either direction. There are equal margins in both directions, indicating that the classification result remains the same, which provides the model basic robustness against normal distribution noise. However, when the image is perturbed by computed noises (b), (c), and (d), the logit output *O* is no longer symmetrical. When *ϵ* increases in L_∞_ norm size, the output *O* decreases more rapidly regarding the correct label, and the output value of the incorrect class may also increase more rapidly. As a result, the incorrect class output value surpasses the correct class output value by a much lesser margin, and the overall classification result is alternated. This observation is consistent within plots (f), (g), and (h); however, the surpass points are different. The surpass point closer to the center zero norm value (marked by the orange line) means that the adversarial noise identified by the algorithm is more severe than others and can alter the output results with a lesser norm size. We marked the FGSM and PGD surpass points with yellow and blue lines that indicate that the FGSM’s adversarial perturbation is less effective than the PGD perturbation. This is expected, since the FGSM is a one-step algorithm that is less powerful than a multi-iteration PGD attack. Our generator’s adversarial surpass point is located between the FGSM and PGD surpass point, which indicates that our generator can find a more effective adversarial perturbation vector than FGSM but that it is still less effective than PGD. This result explains a partial reason as to why our GAN augmentation cannot achieve improved accuracy, since the generator is limited to its capacity.

**Figure 13:**
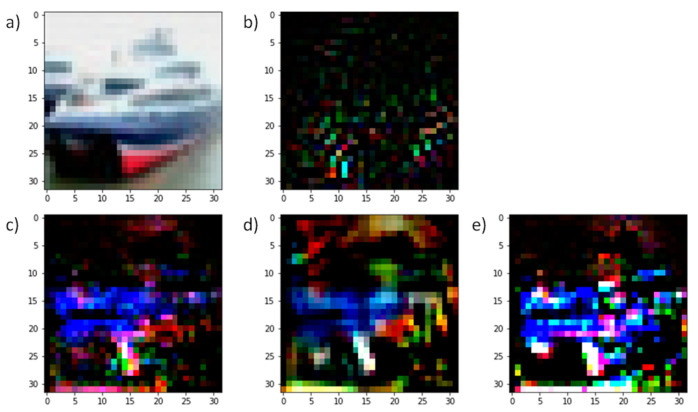
L_2_ adversarial perturbation vectors from different models and attacks. (**a**) The original image, (**b**) the PGD L_2_ perturbation with the benchmark model, (**c**) the PGD L_2_ perturbation with our GAN-trained model, (**d**) Generator’s perturbation, and (**e**) CW-L_2_ adversarial perturbation. All perturbation vectors are scaled up for visibility.

The second group of comparison is between L_2_ constraint adversarial noises from PGD, CW-L_2_, and our generator. L_2_ constraint noise has a different distribution compared to the L_∞_ noises; hence, it is difficult to use the same technique to compare the surpass points under an L_∞_ multiplier. However, we can still demonstrate the difference between the generated noise by comparing the different patterns. Figure 13 displays the visualization of L_2_ noises.

We scaled up the perturbation size for better visibility. Figure 13b indicates the L_2_ PGD perturbation generated based on the benchmark model. Compared to other perturbations, the noise from the benchmark model contains less humanly understandable patterns. Figure 13c,d are generated using our GAN-trained model and exhibit more visible shapes and features from the original image, and the color pattern is more uniform than the noise from the benchmark model. Comparing (c) and (d), it is evident that the noise generated by PGD (c) is sharper and noisier than the perturbation generated by generator (d). However, the generator’s noise has more areas filled with colors. Overall, after GAN training, the adversarial noise is more aligned with human understanding. This result might suggest that the model has learned more robust features with our GAN training. However, the difference between the generated perturbation indicates that there are still some differences between the generator and PGD algorithm that capture different features. We have also presented the adversarial noise from the CW-L_2_ attack. In comparison to PGD L_2_, some features and perturbed pixels are more visible.

### 4.7. Discussion

The experiments in this paper suggest that a classifier can be trained in our proposed generative adversarial network to obtain robustness against gradient-based adversarial attacks. In L_∞_ constraint experiments, we learned that the input formulation of the generator can affect the outcome of the training and can impact the generator’s ability to estimate effective adversarial sample data for augmentation. The gradient vector of the classifier can be the optimal solution to implement as the input vector of the generator compared to other solutions. The highest robust accuracies can achieve around 45% against 8/255 L_∞_ PGD attack and 23% against 16/255 L_∞_ PGD attack. However, the limitation is obvious. First, the current results indicate that the increase in adversarial robustness paired with the penalty in clean accuracy. We referred to this penalty as accuracy–robustness tradeoffs in previous sections, and these tradeoffs appear in all robust classifiers we trained. The average decline of clean accuracy is around 10% to 15% depending on the formulations and parameters. In general, the robustness increase benefit can still outweigh the accuracy decline, especially for the scenarios that required a net increase of both accuracies. However, these tradeoffs hold back the overall generalization of the model. The other effects include overfitting of the classifier and generator. We implemented multiple solutions to mitigate the overfitting effects including the dual generator design, and dropout regularization layers. However, the results suggest that these solutions cannot completely resolve the problem. The other technique, such as weight decay, was also tested in Section 4.1. However, no significant improvement was observed. Second, the generator’s ability to estimate adversarial perturbation must be improved. In the current state, the adversarial noises found by PGD algorithms are more severe than the generator-generated noise. Hence, our classifier can only achieve low robust accuracy under PGD attacks.

In L_2_ constraint settings, we have demonstrated that the L_2_ generator can be a more effective method to augment the data compared to gradient attacks. The L_2_-trained classifier can achieve better accuracy around 60% compared to PGD L_2_ adversarial training around 40% accuracy under 128/255 L_2_ PGD attacks. However, we must note that the robustness increase is only significant in gradient-based attack algorithms. Attacks using other optimization methods might result in different adversarial sample solutions. We cannot guarantee that the model is robust under different solutions. The results suggest that PGD L_2_ attack can only provide an unconvincing assessment on L_2_ robustness. However, the results still demonstrate that our proposed GAN is more effective compared to PGD L_2_ adversarial training. We also suggest that the current non-robust results might be caused by our classifier’s capability. Therefore, more experiments are required to draw further conclusions. Furthermore, the L_2_ generator-trained model indicates nearly identical robustness against L_∞_ constraint PGD adversarial samples compared to the L_∞_ generator-trained model. We suggest that these two types of generators can provide robustness for different constraints but only with finetuned scalar values.

Ultimately, the difference in adversarial noise generated with the baseline and our GAN-trained model demonstrates a different learned data distribution. The limitation is that by learning this different data distribution, the clean accuracy is also affected. The GAN-trained model overfitted strongly to the new data distribution and cannot provide the same generalization to all the data points. More research is required to remove the unexpected data shift, which might relate to bias introduced with our GAN generator. 

Additionally, our generator had a similar performance in attacking our classifier model compared to the CW-L_2_ attack. Previously, it was suggested that gradient-based attacks might suffer from gradient masking problems [2] with L_2_ constraint. We suggest that using our suggested GAN formulation can overcome this problem to some degree. It can effectively learn the perturbation from its co-trained classifier and perform an effective attack. 

## 5. Conclusions and Future Work

This paper introduced a GAN-based model to augment training data to improve a classifier’s L_∞_ and L_2_ adversarial robustness against gradient-based attacks. The proposed GAN-based model was successfully implemented with dual generator architecture, varieties of input formulations, and different constraint formulations. The experiment suggests that the model successfully gains robustness under the different settings. However, the generalization of the model is not guaranteed. The clean accuracy declines after training, and the robustness cannot be applied against other types of attacks. To further verify the possibility of this framework, we need to implement and evaluate a larger model with more parameters. We hope that by increasing the model’s capacity, we can further improve the robustness against other adversarial samples. However, we wish to highlight the performance of the GAN generator in finding adversarial perturbation. We suggest that it can be another efficient and effective method to obtain the adversarial samples.

In the image classification model, selecting the distance constraints has always been challenging for adversarial machine learning. The L_∞_ constraint is usually more practical to limit the visible changes within the image. However, we still need to discover new conceptual distance metrics that align more with our human understanding. With this GAN model, we plan to expand it to make it work on different constraints simultaneously. We hope there is some way to provide model robustness on a universal constraint. We also plan to apply the proposed model to other application domains, as GANs can be used to augment data for time series and tabular data.

## Figures and Tables

**Table 1:** Related work comparison.

Defensive Training Methods	Related Papers	Benefits	Limitations
Conventional adversarial training	Baseline [2,6]	More reliable than GAN approaches Simple formulation	Training complexity Requirement to implement a specific attack algorithm
GAN cleanse model	[8,9,10,11]	Transferable for different classifier	Including additional model deployment in application Required adversarial attacks to generate training samples and requiring more training complexity The performance is related to the model’s implementation No robustness improvements on original classifier
GAN reconstruction model	[12,13,14,15,16]	Transferable for different classifier Not requiring adversarial attack implementation (attack independent)	Including additional model deployment in application The performance is related to the model’s implementation No robustness improvements on original classifier
GAN improved adversarial training frameworks	[17,18]	Improving conventional adversarial training	The performance is related to model implementation details
GAN adversarial augmentation frameworks	[19]	Reducing training complexity Not requiring attack algorithm implementation (attack independent)	The performance is related to model implementation details
Our proposal: an improved GAN adversarial augmentation method		Retaining all of the benefits from GAN adversarial augmentation frameworks Proposing new formulations for GAN that improves the adversarial sample estimation and robust accuracies. The new formulations include a dual generator architecture, four generator input formulations, and L_∞_ and L_2_ generator constraints implementations. Providing additional information related to the limitations of our GAN, including accuracies tradeoffs, training epoch’s impact, overfitting of our GAN, and limited generalization capabilities. Suggesting a direction of future improvement	

**Table 2:** L_∞_ generator filter parameters.

Output Size	Generator Filters (×Width)
32 × 32 × 32	7 × 7, 32, stride 1
16 × 16 × 64	5 × 5, 64 × width, stride 2
8 × 8 × 128	5 × 5, 128 × width, stride 2
8 × 8 × 128	$\left[\begin{array}{c} 3\times 3,\;128\times \mathrm{width}\\ 3\times 3,128\times \mathrm{width} \end{array}\right]\times 4$
16 × 16 × 64	5 × 5, 64 × width, stride 2
32 × 32 × 32	5 × 5, 32 × width, stride 2
32 × 32 × 3	7 × 7, 3, stride 1

**Table 3:** L_2_ generator filter parameters.

Output Size	Generator
32 × 32 × 32	[1 × 1, 32] × 1 $\left[\begin{array}{c} 3\times 3,\;32\\ 3\times 3,\;32 \end{array}\right]\times 2$
16 × 16 × 32	Average pooling
16 × 16 × 64	[1 × 1, 64] × 1 $\left[\begin{array}{c} 3\times 3,\;64\\ 3\times 3,\;64 \end{array}\right]\times 2$
8 × 8 × 64	Average pooling
8 × 8 × 96	[1 × 1, 96] × 1 $\left[\begin{array}{c} 3\times 3,\;96\\ 3\times 3,\;96 \end{array}\right]\times 2$
4 × 4 × 96	Average pooling
4 × 4 × 128	[1 × 1, 128] × 1 $\left[\begin{array}{c} 3\times 3,\;128\\ 3\times 3,\;128 \end{array}\right]\times 2$
8 × 8 × 128	Up sampling
8 × 8 × 64	[1 × 1, 96] × 1 $\left[\begin{array}{c} 3\times 3,\;96\\ 3\times 3,\;96 \end{array}\right]\times 2$
16 × 16 × 64	Up sampling
16 × 16 × 32	[1 × 1, 64] × 1 $\left[\begin{array}{c} 3\times 3,\;64\\ 3\times 3,\;64 \end{array}\right]\times 2$
32 × 32 × 32	Up sampling
32 × 32 × 32	[1 × 1, 32] × 1 $\left[\begin{array}{c} 3\times 3,\;32\\ 3\times 3,\;32 \end{array}\right]\times 2$
32 × 32 × 3	[1 × 1, 3] × 1

**Table 4:** Classifier filter parameters.

Output Size	Classifier
32 × 32 × 32	[3 × 3, 32] × 2
16 × 16 × 32	Max pooling
16 × 16 × 64	[3 × 3, 64] × 2
8 × 8 × 64	Max pooling
8 × 8 × 128	[3 × 3, 128] × 2
4 × 4 × 128	Max pooling
4 × 4 × 256	[3 × 3, 256] × 2
2 × 2 × 256	Max pooling
1024	Flatten
10	Dense (10)

**Table 5:** L_∞_ PGD 100 and 1000 accuracies.

**L_∞_ Norm Size**	4/255	8/255	16/255
**Accuracy PGD 100**	57.41%	45.6%	23.71%
**Accuracy PGD 1000**	57.33%	45.28%	23.31%

**Table 6:** Accuracy comparison under different attacks.

PGD L_2_ (64/255)	CW-L_2_ (Non-Constraint)	Generator (64/255)
68.73%	20.58%	22.00%

## Data Availability

Not applicable.
